# Factors predictive of relapse in adult bacterial osteomyelitis of long bones.

**DOI:** 10.1186/s12879-018-3550-6

**Published:** 2018-12-07

**Authors:** E. Garcia del Pozo, J. Collazos, J. A. Carton, D. Camporro, V. Asensi

**Affiliations:** 1 Plastic Surgery Service, Hospital Universitario Central de Asturias (HUCA), Oviedo, Asturias Spain; 2 Infectious Diseases Unit, Hospital de Galdácano, Galdacano, Vizcaya Spain; 3 Infectious Diseases Unit, Hospital Universitario Central de Asturias (HUCA), Oviedo University School of Medicine, Oviedo, Spain; 4 Group of Translational Research in Infectious Diseases, Instituto de Investigación Sanitaria del Principado de Asturias (ISPA), Asturias, Spain

**Keywords:** Osteomyelitis, Relapse, Severity factors, Debridement, Muscular flap, Antibiotics

## Abstract

**Background:**

Osteomyelitis is a difficult-to-cure infection with a high relapse rate despite combined medical and surgical therapies. Some severity factors, duration of antimicrobial therapy and type of surgical procedure might influence osteomyelitis relapse.

**Methods:**

116 patients with osteomyelitis were followed for ≥1 year after hospital discharge. Demographic, microbiological and clinical data, eight severity factors and treatment (surgical and antibiotic) were analyzed.

**Results:**

Mean age was 53 years and 74.1% were men. Tibia (62.1%) and *S. aureus* (58.5%) were the most commonly involved bone and bacteria, respectively. Mean follow-up was 67.1 months. Forty-six patients underwent bone debridement, 61 debridement plus flap coverage and 9 antimicrobial therapy only. Twenty-six patients (22.4%) relapsed, at a mean of 11.2 months since hospital discharge. Duration > 3 months (*p* = 0.025), number of severity factors (*P* = 0.02) and absence of surgery (*P* = 0.004) were associated with osteomyelitis relapse in the univariate analysis. In the Cox regression analysis, osteomyelitis duration > 3 months (*P* = 0.012), bone exposure (*P* = 0.0003) and type of surgery (*P* < 0.0001) were associated with relapse. Regarding the surgical modalities, bone debridement with muscle flap was associated with better osteomyelitis outcomes, as compared with no surgery (*P* < 0.0001) and debridement only (*P* = 0.004).

**Conclusions:**

Osteomyelitis extending for > 3 months, bone exposure and treatment other than surgical debridement with muscular flap are risk factors for osteomyelitis relapse.

## Background

Osteomyelitis is a difficult-to-treat bone infection characterized by progressive and inflammatory destruction of the infected bone and new apposition of bone at the site of infection. In adults, osteomyelitis is usually a complication of open wounds due to fractures, surgery, or both. The risk and severity of the infection can be enhanced by the presence of foreign bodies (metallic or prosthetic devices). It is reported that 0.4 to 7% of trauma and orthopedic interventions are complicated by osteomyelitis [1–6]. This infection can develop as well in non-injured bones after bacteremia, mostly in prepubertal children and in elderly patients, in which the infection involves mostly the axial skeleton. *Staphylococcus aureus* is the microorganism most frequently isolated in both posttraumatic and hematogenous osteomyelitis. Despite appropriate combined medical and surgical therapies, recurrences are common, often in the range of 20–30%, causing significant morbidity and mortality, as well as major economic losses [7–11].

The features associated with osteomyelitis relapse, as well as their relative weights have not been clearly demonstrated so far, and their identification might be important in order to optimize antimicrobial and surgical therapies in those patients prone to relapse. Among these features, a number of demographic, laboratory, clinical, microbiological and therapeutic, both antibiotic and surgical, factors might be potentially involved. On the other hand, the identification of possible factors of severity at the time of presentation that could influence the outcome would be highly desirable. However, these factors remain largely unknown.

Therefore, the aim of this study was to evaluate the parameters associated with relapse in patients with osteomyelitis of long bones from a single center and, particularly, to evaluate the possible influence on the outcome of certain, predefined severity factors.

## Patients and methods

### Patients

Adult patients with a diagnosis of bacterial osteomyelitis of the long bones, who were admitted to the Hospital Universitario Central de Asturias (HUCA) between January 1st 1994 and October 1st 2015, and who were followed-up for at least one year after discharge, were included in the study. HUCA is a 900 bed, third-level academic hospital that provides health coverage to one million people from the region of Asturias, Northwestern Spain. Open bone fractures of long bone were classified according to Gustilo-Anderson [12].

Osteomyelitis was diagnosed using clinical and roentgenographic findings. The demonstration of bone sequestra and/or sinus tract in bone X-ray, computed tomography (CT), or magnetic resonance imaging (MRI), a positive Ga^67^ uptake bone scan, or a positive culture of the sequestra or sinus tract were considered diagnostic of osteomyelitis [3–7]. Osteomyelitis patients with no history of trauma or bone surgery, no lower limb vascular insufficiency, and without a contiguous focus of infection were considered to have hematogenous osteomyelitis.

Patients with osteo-articular tuberculosis were excluded. Osteomyelitis was considered cured if no relapse was detected during the follow-up. Surgical and sinus tract pus samples were cultured and erythrocyte sedimentation rate (ESR) and C-reactive protein (C-RP) were also recorded. A number of demographic, clinical, microbiological and therapeutic data, including the type and duration of antibiotic therapy and the modality of surgical procedure used, were also collected. Eight predefined local and systemic severity factors for osteomyelitis were considered: relapse of prior osteomyelitis, duration > 3 months, presence of osteosynthesis material, bone exposure, peripheral vascular involvement, diabetes, multiresistant/polymicrobial infection and immunosuppression. All the study data were introduced into a database and analyzed from October 2016 onwards, one year after the ending of the inclusion period.

### Statistical analysis

Continuous variables are reported as mean (95% CI) and categorical variables as n (%). The comparisons between the relapse and non-relapse cases were carried out by the t-test and the chi-square or Fisher exact tests when appropriate, for continuous and categorical variables, respectively. Kaplan-Meier survival curves were used to evaluate the relationships of the different severity factors with the outcome, and their statistical significance was assessed by means of the log-rank test. A stepwise logistic regression analysis was carried out to evaluate the relative importance of each severity factor, in order to elaborate a predictive formula of the outcome. The result of this formula was tested by means of a receiver operating characteristic (ROC) curve, and the area under the ROC curve was also calculated. A stepwise Cox regression analysis was constructed to identify the variables independently associated with the relapse of osteomyelitis. SPSS v.22 software was used for statistical calculations. A *P* value < 0.05 for a two-sided test was considered statistically significant.

## Results

A total of 116 patients with osteomyelitis of long bones of the arms and legs, were analyzed. The mean age was 53.0 years (95% CI 49.8–56.2) and 86 of them (74.1%) were men. Osteomyelitis was secondary to open fractures in 54 (46.6%) patients: Gustilo-Anderson type I 18 (33.3%), type II a 7 (13.0%), type II b 24 (44.4%) and III c 5 patients (9.3%). The mean period of follow-up was 67.1 months (95% CI 57.3–76.9 months). During this period there were 26 relapses (22.4%), which occurred at a mean of 11.2 months (95% CI 3.3–19.1 months) since hospital discharge. Among the relapses 6 (23.1%) might be treatment failures. Although the attending doctor considered these 6 osteomyelitis cases as cured, a clear disappearance of clinical symptoms and/or roentgenographic findings and/or normalized C-RP and ESR values at the end of therapy was not registered in the medical charts. Of the remaining 20 patients a relapse was registered within the first year after finishing therapy in 12 (46.2%) and more than one year after in other 8 patients (30.8%)*.*

The bone most commonly involved was the tibia (62.1% of cases), and the most commonly isolated bacteria was *Staphylococcus aureus* (58.5% of the positive cultures). Imaging studies other than simple radiographs were used in 56.3% of the patients, C-RP was elevated in 43.2% and ESR in 89.4% of the osteomyelitis patients. The time elapsed since diagnosis and treatment until the evaluation in October 2016 did not have any influence on the outcome (*P* = 0.8).

Table 1 shows the demographical, clinical, microbiological and therapeutic features of the osteomyelitis cases. There were significant differences between the patients who relapsed and those who did not relapse regarding the duration of symptoms, number of severity factors, and absence of surgical treatment. On the contrary, there were no significant differences in aspects such as demography, source of infection, bones involved, acute phase reactants, microbiology or type or duration of antibiotic treatment. No differences in relapse rate between those with methicillin-resistant (MRSA) and methicillin-susceptible *Staphylococcus aureus* (MSSA) osteomyelitis were observed either (data not shown in Table 1).

**Table 1:** Demographic, clinical, microbiological and therapeutic features of the patients according to the outcome

		Relapse (*n* = 26)	No relapse (*n* = 90)	P
Gender	Male	16 (18.6%)	70 (81.4%)	0.096
	Female	10 (33.3%)	20 (66.7%)	
Age	Years	57.42 (51.13–63.72)	51.67 (47.93–55.40)	0.14
Origin/source	Hematogenous	4 (20.0%)	16 (80.0%)	0.5
	Post-traumatic	12 (19.4%)	50 (80.6%)	
	Post-surgical	10 (29.4%)	24 (70.6%)	
Bones involved	Femur	6 (22.2%)	21 (77.8%)	0.9
	Tibia	17 (23.6%)	55 (76.4%)	
	Fibula	1 (16.7%)	5 (83.3%)	
	Humerus	0 (0%)	3 (100%)	
	Ulna	1 (20.0%)	4 (80.0%)	
	Radius	1 (33.3%)	2 (66.7%)	
Imaging (other than radiographs)	Yes	20 (28.2%)	51 (71.8%)	0.06
	No	6 (13.3%)	39 (86.7%)	
C Reactive Protein (C-RP)	mg/L	8.865 (.357–17.373)	6.440 (4.739–8.1395)	0.6
Erythrocyte sedimentation rate (ESR)	mm/h	53.52 (37.26–69.78)	65.01 (57.20–72.82)	0.18
Duration of follow-up	Months	56.12 (37.80–74.43)	70.28 (58.67–81.89)	0.24
Severity factors				
Relapse of previous osteomyelitis	Yes	14 (28.6%)	35 (71.4%)	0.17
	No	12 (17.9%)	55 (82.1%)	
Symptoms longer than 3 months	Yes	22 (28.6%)	55 (71.4%)	0.025
	No	4 (10.3%)	35 (89.7%)	
Presence of osteosynthesis material	Yes	13 (22.0%)	46 (78.0%)	0.9
	No	13 (22.8%)	44 (77.2%)	
Bone exposure	Yes	12 (26.1%)	34 (73.9%)	0.4
	No	14 (20.0%)	56 (80.0%)	
Vascular involvement	Yes	5 (45.5%)	6 (54.5%)	0.067
	No	21 (20.0%)	84 (80.0%)	
Diabetes	Yes	3 (30.0%)	7 (70.0%)	0.7
	No	23 (21.7%)	83 (78.3%)	
Multiresistant/polymicrobial infection	Yes	12 (23.5%)	39 (76.5%)	0.8
	No	14 (21.5%)	51 (78.5%)	
Immunosuppression	Yes	2 (50.0%)	2 (50.0%)	0.22
	No	24 (21.4%)	88 (78.6%)	
Presence of severity factors	Yes	12 (29.3%)	29 (70.7%)	0.19
	No	14 (18.7%)	61 (81.3%)	
Types of severity factors	No	14 (18.7%)	61 (81.3%)	0.4
	Local	5 (22.7%)	17 (77.3%)	
	Systemic	4 (40.0%)	6 (60.0%)	
	Both	3 (33.3%)	6 (66.7%)	
Number of severity factors		3.19 (2.69–3.69)	2.49 (2.2–2.76)	0.02
Microbiology				
Gram-positive cocci ^a^	Gram-positive cocci	17 (24.6%)	52 (75.4%)	0.8
	Others	6 (27.3%)	16 (72.7%)	
Gram-negative bacilli ^a^	Gram-negative bacilli	9 (28.1%)	23 (71.9%)	0.8
	Others	15 (25.4%)	44 (74.6%)	
*Staphylococcus aureus* ^a^	*S aureus*	12 (25.0%)	36 (75.0%)	0.9
	Others	8 (23.5%)	26 (76.5%)	
Gram-pos cocci vs Gram-neg bacilli ^a^	Gram-positive cocci	14 (25.0%)	42 (75.0%)	0.8
	Gram-negative bacilli	6 (27.3%)	16 (72.7%)	
Treatment				
Type of treatment	Antibiotics only	6 (66.7%)	3 (33.3%)	0.004
	Antibiotics plus surgery	20 (18.7%)	87 (81.3%)	
Intravenous beta-lactams	β-lactams	5 (13.9%)	31 (86.1%)	0.7
	Other antibiotics	4 (18.2%)	18 (81.8%)	
Oral β-lactams	β-lactams	4 (23.5%)	13 (76.5%)	0.7
	Other antibiotics	9 (17.3%)	43 (82.7%)	
Oral clindamycin	Clindamycin	3 (10.7%)	25 (89.3%)	0.21
	Other antibiotics	11 (22.0%)	39 (78.0%)	
Oral rifampin	Rifampin	4 (15.4%)	22 (84.6%)	0.8
	Other antibiotics	10 (19.2%)	42 (80.8%)	
Duration of intravenous antibiotics	Days	26.44 (23.24–29.64)	29.33 (27.09–31.58)	0.21
Total duration of antibiotic treatment	Days	80.76 (36.05–125.47)	69.58 (62.66–76.49)	0.40
Type of surgery	None	6 (66.7%)	3 (33.3%)	0.004
	Debridement	9 (19.6%)	37 (80.4%)	
	Debridement and flap	11 (18.0%)	50 (82.0%)	
Removal of osteosynthesis material ^b^	Yes	8 (19.0%)	34 (81.0%)	0.24
	No	4 (36.4%)	7 (63.6%)	
Type of surgical reconstruction	None	15 (27.3%)	40 (72.7%)	0.6
	Free muscle flap	2 (14.3%)	12 (85.7%)	
	Pedicled muscle flap	4 (19.0%)	17 (81.0%)	
	Free fasciocutaneous flap	5 (23.8%)	16 (76.2%)	
	Pedicled fasciocutaneous flap/rotation	0 (0%)	5 (100%)	
Surgical reconstruction	Yes (all types of flaps)	11 (18.0%)	50 (82.0%)	0.23
	No	15 (27.3%)	40 (72.7%)	

Values are expressed as mean (95% CI) or n (%) as appropriate

^a^The comparisons of microorganisms/antibiotics have been carried out only in the cases in which the bacteria/antibiotic have been identified

^b^Only in patients who had osteosynthesis material

**Fig. 1:**
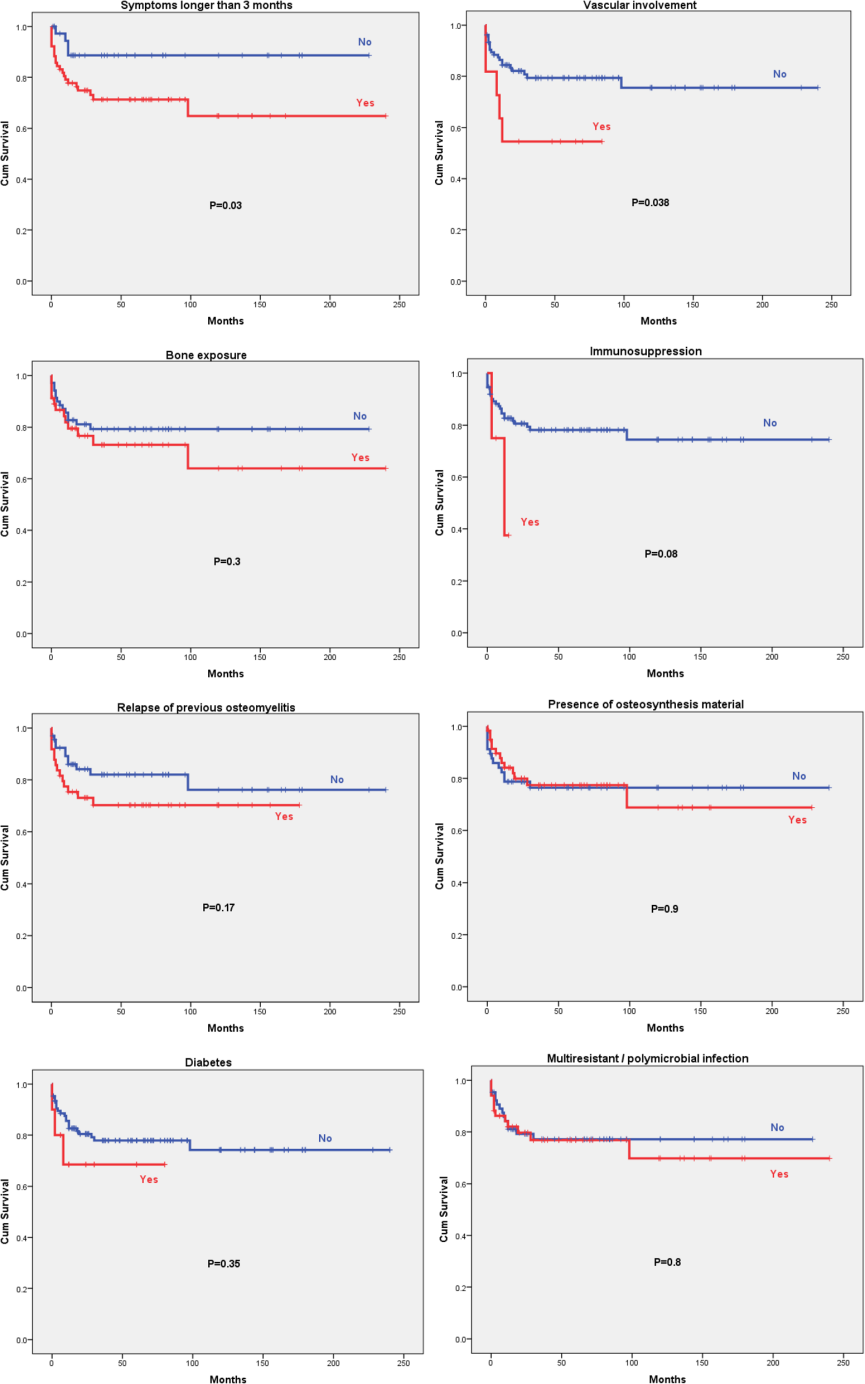
Kaplan Meier cumulative survival curves of the eight severity factors for osteomyelitis

Figure 1 shows the Kaplan Meier survival curves for each of the 8 severity factors evaluated. A logistic regression analysis was carried out to construct a formula in order to predict the risk of relapse based solely on the severity factors studied. The resulting formula was: 1.5 points (if the patient had symptoms for longer than 3 months) + 0.5 points (if there was bone exposure) + 1.2 points (if there was peripheral vascular involvement) + 2.0 points (if the patient was immunosuppressed) + 0.2 points (if there was osteosynthesis material).

**Fig. 2:**
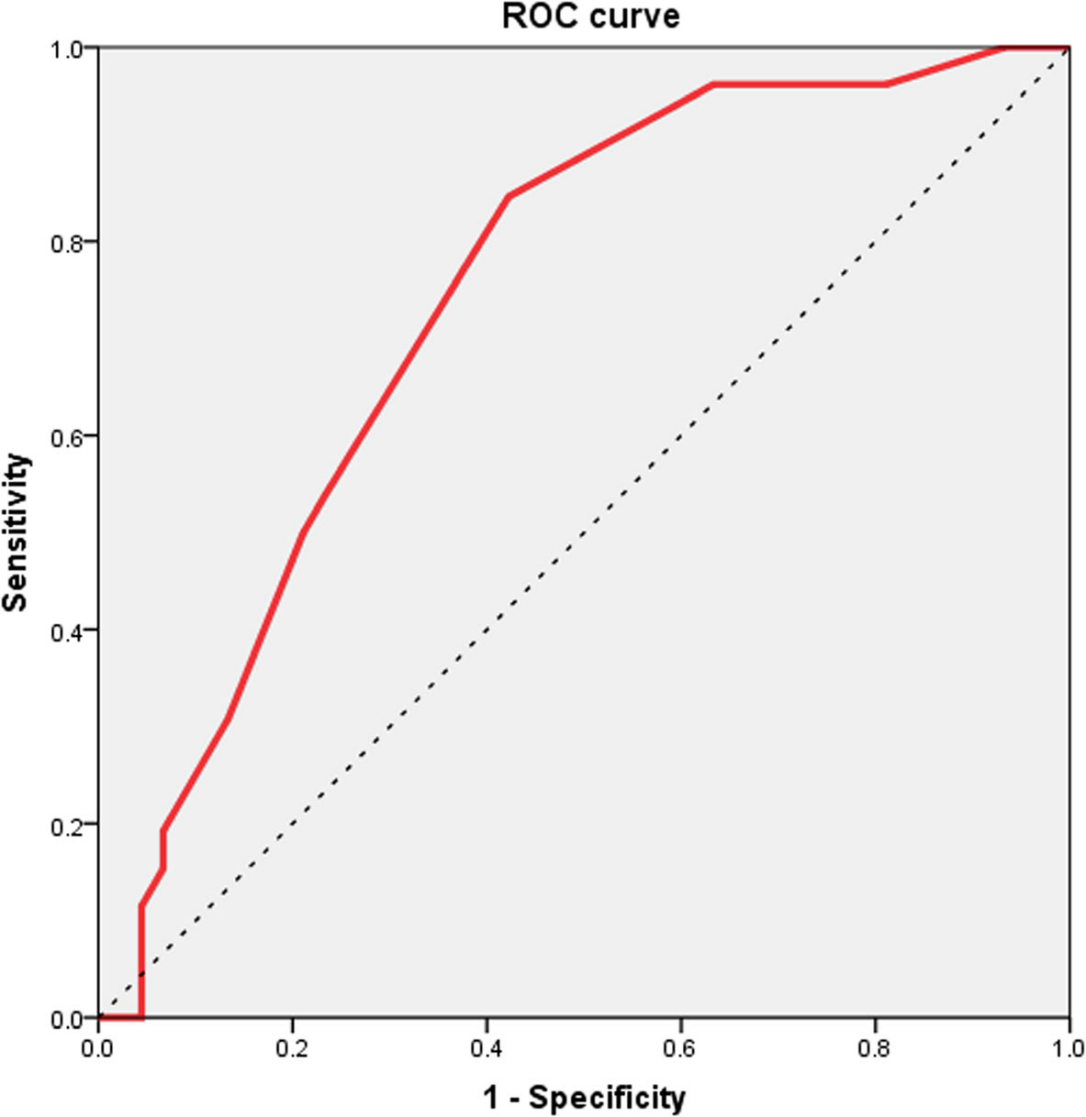
Receiver operating characteristic (ROC) curve corresponding to the predictive formula

Figure 2 depicts the ROC curve for the result of the application of that formula. The application of that formula differentiates relapsing and not relapsing patients with different sensitivities and specificities depending on the cut-off used. Higher cut-offs are associated with progressively higher sensitivities and lower specificities, whereas the opposite occurs in the case of lower cut-offs. Figure 2 depicts the resulting ROC curve which had an area under the curve was of 0.742 (95% CI 0.644–0.839, *P* = 0.0002). According to this formula a score of ≥1.5 points would be associated with a probability of relapse of 30.5% (positive likelihood ratio 1.52, 95% CI 1.28–1.81), a score of ≥2 points with a probability of 40.6% (positive likelihood ratio 2.37, 95% CI 1.52–3.69) and a score of ≥2.5 with a probability of 45.5% (positive likelihood ratio 2.88, 95% CI 1.31–6.34).

The area under the ROC curve was 0.742 (95% CI 0.644–0.839), *P* = 0.0002). According to this formula a score of ≥1.5 points would be associated with a probability of relapse of 30.5% (positive likelihood ratio 1.52, 95% CI 1.28–1.81), a score of ≥2 points with a probability of 40.6% (positive likelihood ratio 2.37, 95% CI 1.52–3.69) and a score of ≥2.5 with a probability of 45.5% (positive likelihood ratio 2.88, 95% CI 1.31–6.34).

A Cox regression model was constructed using the variables with a *P* value ≤0.2 in the univariate analysis, as well as others of clinical relevance, to identify the factors independently associated with the outcome of the osteomyelitis over time. According to this model, the variables predictive of relapse were: duration of symptoms longer than 3 months, bone exposure and type of surgical therapy. 

**Fig. 3:**
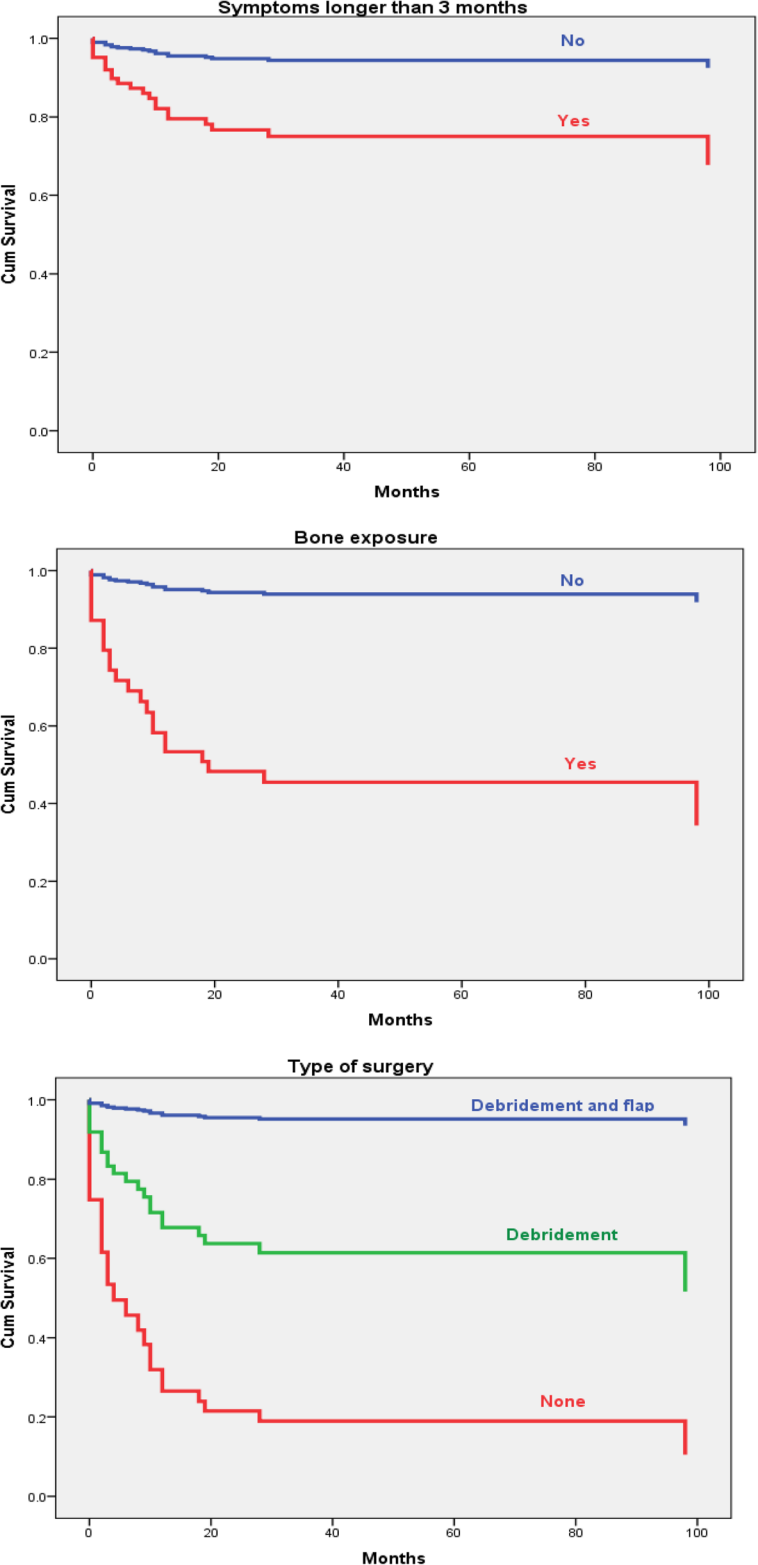
Variables independently associated with osteomyelitis relapse in the Cox regression analysis

Among the latter, the combined use of antibiotics plus debridement and flap coverage was the most successful modality of treatment, whereas the absence of surgical treatment was predictive of the poorest outcome (Table 2 and Fig. 3).

**Table 2:** Variables significantly associated with relapse according to the Cox regression analysis

	HR (95% CI)	P
Symptoms longer than 3 months	4.98 (1.42–17.54)	0.012
Bone exposure	12.66 (3.15–49.26)	0.0003
Type of surgical treatment		< 0.0001
None vs Debridement	3.41 (1.12–10.42)	0.031
None vs Debridement and flap	33.27 (7.80–141.88)	< 0.0001
Debridement vs Debridement and flap	9.75 (2.10–45.21)	0.004

On the contrary, there were no statistical significant associations regarding gender (*P* = 0.056), age (*P* = 0.4), relapse of previous osteomyelitis (*P* = 0.8), presence of osteosynthesis material (*P* = 0.12), peripheral vascular involvement (*P* = 0.8), diabetes (*P* = 0.7), multiresistant bacteria or polymicrobial infection (*P* = 0.6), immunosuppression (*P* = 0.9), ESR (*P* = 0.3), imaging other than simple radiographs (*P* = 0.5), duration of intravenous antibiotic therapy (P = 0.5), total duration of antibiotic treatment (*P* = 0.9) or time elapsed since diagnosis (*P* = 0.9).

## Discussion

We have analyzed the factors involved in the relapse of bacterial osteomyelitis of long bones in our series. Bone infection relapsed in 20.6% of the patients followed for more of one year, a slightly lower rate compared to that found in previous reports of adult osteomyelitis [6–11]. The parameters independently associated with osteomyelitis relapse in the multivariate analysis were duration of the infection for more than 3 months at presentation, bone exposure, mostly after an open fracture, and treatment other than surgical debridement with flap coverage. We have also developed a mathematical formula based on the predefined severity factors, and observed that the probability of relapse increased accordingly, being of 30.5, 40.6 and 45.5% for scores ≥1.5, ≥2, and ≥ 2.5, respectively.

To our knowledge, only few clinical series focusing on the risk factors and outcome of bacterial osteomyelitis have been reported so far, one is a pediatric series of 83 children [13] while four other series included over 100 adult osteomyelitis patients each [8, 9, 11, 14] (Table 3). Ours is the second work in which a score made of the addition of clinical elements or points to predict osteomyelitis outcome is used and the only one in which this score is applied to adults, focuses exclusively on long bone osteomyelitis of different pathogenesis, analyzes relapses or is derived from the outcome. In fact. Roine’s osteomyelitis score was applied to children with acute hematogenous osteomyelitis mostly, but not exclusively, of the long bones, focused to the development of diverse sequelae, not to relapses, and was pre-established, not derived from true results [13]. These authors considered as factors associated with osteomyelitis sequelae in children the following: axillary temperature > 37.4 °C for > 7 days, marked local swelling or warmth for > 10 days, marked local pain or limited motility for > 10 days, additional surgical drainage after the initial one and more than one focus of osteomyelitis or septic shock in addition to high ESR and C-RP, and assigned 1 point to each of these pre-established factors. On the contrary, we calculated the relative weight of the different severity factors according to the outcome.

**Table 3:** Comparison of the demographic and clinical characteristics and recurrence factors in clinical studies of osteomyelitis

		Roine [13] 1996	Tice [8, 9] 2003	Arias [11] 2015	Lin [14] 2016	Present series 2017
No. patients (% males)		83 (NS)	454 (65%)	129 (80.6%)	108 (75%)	116 (74.1%)
Age group		Children (mean 6.7 y)	Mostly adults (mean 51 y)	Adults (range 18–91 y)	Mostly adults (median 54 y)	Adults (mean 53 y)
Country		Costa Rica	USA	Colombia	Singapore	Spain
Origin		Hematogenous	Multiple	Multiple	Multiple	Multiple
Post-traumatic (n, %)		0 (0%)	409 (90.1%) ^a^	115 (89.2%)	42 (38.9%)	62 (53.4%)
Bones involved		Mostly long bones	Mostly non-long bones	Mostly long bones	Mostly long bones	Only long bones
*S. aureus* (n, %) ^b^		41 (49.4%)	246 (54.2%)	49 (38.0%)	42 (38.9%)	48 (38.1%)
Diabetes		NS	173 (38.1%)	7 (5.4%)	58 (53.7%)	10 (8.6%)
Recurrence (n, %)		5 (6.0%)	139 (30.6%)	30 (23.3%)	40 (37.0%)	26 (20.6%)
Mean follow-up (months)		1.5 or 14 ^c^	28.3	12	23,4	67.1
Factors associated with recurrence	Microorganisms involved	*S aureus* ^d^	*P aeruginosa*	No	MRSA	No
	Antibiotic type	No ^d^	Yes (vancomycin) ^e^	No	NS	No
	Antibiotic duration	No ^d^	NS	No	No	No
	Age	No ^d^	No (> 70 y)	No	No	No
	Diabetes	NS ^d^	Yes	No	No	No
	Peripheral vascular disease	NA ^d^	Yes	No	No	No
	Long bones	NS ^d^	NS	No	Yes (lower limbs)	No
	Fracture	NA ^d^	NS	No	No	No
	Symptoms duration	No ^d^	NS	NS	NS	Yes (> 3 months)
	Bone exposure	NA ^d^	NS	No	No	Yes
	Type of surgery	No ^d^	NS	NS	No	Yes
	C-RP	Yes ^d^	NS	NS	No	No
	ESR	No ^d^	NS	NS	Yes (≥ 20 mm/h)	No

*NA* denotes not applicable, *NS* not studied, *MSSA* methicillin-susceptible *Staphylococcus aureus*, *MRSA* methicillin-resistant *Staphylococcus aureus*, *C-RP C*-reactive protein, *ESR* erythrocyte sedimentation rate

^a^Described as associated with wounds

^b^Respect to the total number of patients

^c^All patients were followed-up for a mean of at least 1,5 months, and 78% of them for a mean of 14 months

^d^Factors associated with sequelae (28 patients, 33.7%); data for recurrence are not provided

^e^Only in staphylococcal infections

Our study includes adults with osteomyelitis of the long bones with a post-traumatic or post-surgical source in 82.8% of the cases. Therefore, we analyzed the presence of osteosynthesis material and bone exposure, because most of our patients suffered accidental fracture or surgical bone damage preceding the infection. In addition peripheral vascular involvement, diabetes and immunosuppression that we analyzed as potential osteomyelitis recurrence factors are rarely or not observed in children. In this regard, Tice et al [8, 9] found that peripheral vascular disease and/or diabetes were associated with osteomyelitis recurrence in a large series of mostly adult patients, with osteomyelitis mainly of the feet, hand and spine, and who also had a high prevalence of diabetes (38.1%) (Table 3). We did not find such an association in our series composed of a relatively low number of patients with these two conditions (< 10%) and that excluded osteomyelitis of the feet, commonly seen in diabetic patients. On the other hand, Arias et al [11] found in a recent series of 129 osteomyelitis patients (91 with long-bone involvement) that the only factor significantly associated with recurrence was the specialty of the treating clinician (orthopedics as compared to infectologists).

Regarding the microorganism involved, *P aeruginosa* [9] and MRSA or MSSA [13–17] have been associated with worse osteomyelitis outcomes. However, similarly to other authors that analyzed mainly long-bone osteomyelitis [11], we failed to find any association between any bacteria and osteomyelitis outcome.

In our study, bone debridement with flap coverage was associated with better osteomyelitis outcomes as compared to no surgery or exclusive debridement. The use of local muscle flaps in the treatment of osteomyelitis has been reported long ago to cover a large defect of soft tissue and bone, after debridement, with a success rate of above 95% [18, 19]. Nowadays it is known that there are no differences between muscle and perforator fasciocutaneous flaps vascularization, and both of them, or even bone flaps, might be used for reconstruction in osteomyelitis after wide debridement.

Because there is a great variety of flaps, the election of muscle or fasciocutaneous, pedicled or free flaps must be made according to the characteristics of the defect to cover, of the recipient and of donor site morbidity [20–26]. We analyzed 61 osteomyelitis patients treated with different types of flap coverage, and we did not observe differences regarding osteomyelitis relapse among the different flap modalities (*P* = 0.6), although any type of flap coverage, in addition to bone debridement, resulted in better osteomyelitis outcomes, as compared with no surgery (*P* < 0.0001) and bone debridement only (*P* = 0.004). However, there are other promising techniques that were not used in our series, but could be applied for filling bone defects instead of, or in combination with, flaps like the use of bone substitutes, which have had a great development in the last years and could be used in association with locally-liberated antibiotics No studies comparing the results of bone substitutes vs. flap coverage have been reported so far but its combined use might reduce donor flap site morbidity and even avoid further surgical procedures Therefore, these promising techniques should be taken into account in the surgical management of osteomyelitis and bone reconstruction [27–29].

The optimal duration of antibiotic treatment in osteomyelitis still remains unclear. Traditionally osteomyelitis has been treated with 4–6 weeks of parenteral antibiotics after definitive debridement surgery [4–7]. A review of the literature regarding the ideal duration of parenteral antimicrobial therapy concluded that the critical point regarding cure or recurrence relies more on the surgical technique than on the antimicrobial time frame [30]. Also, there is no evidence that prolonged parenteral antibiotics will improve the penetration in the necrotic bone. However, a vascularized flap would allow the establishment of better bone blood supply and better antibiotic release in the infected bone, allowing a shorter duration of treatment. An adequate surgical approach.

combining complete debridement and a well-vascularized flap coverage, both factors associated with better outcomes in our series, would facilitate shorter intravenous therapies, even of only 2 weeks [30–32].

The type of antimicrobials used, and the duration of intravenous or combined oral and intravenous therapies did not associate with osteomyelitis relapse in our study. However, it should be considered that the mean duration of intravenous therapy in our series (about 4 weeks) was similar in both groups and large enough to ensure sufficient antibiotic therapy. Therefore, any possible impact of treatment duration on the outcome would be minimized. Consequently, we cannot completely exclude that shorter periods of treatment could have an influence on the relapse rate. On the other hand, the similarity between the two groups regarding the duration of antibiotic therapy contributes to exclude this potentially important interacting factor and to stress the value of the other factors associated with relapse found in our study.

Our osteomyelitis series differs in several facts from those previously published (Table 3). It includes a large number of cases of bacterial osteomyelitis mostly of post-traumatic or post-surgical origin, limited exclusively to long bones of adults, with the longest follow-up of those published (mean 67.1 months), and it is also the only one in which different surgical approaches to osteomyelitis are compared. In addition, we bring forward here an osteomyelitis recurrence score that might be helpful to the clinicians involved in the care of these patients to predict at presentation the risk of relapse.

Apart from symptoms duration and bone exposure, we did not find that other severity factors considered individually were significantly associated with relapse, such as the presence of osteosynthesis material, vascular compromise, diabetes, multiresistant or polymicrobial infection or underlying immunosuppression, although the presence of at least some of them seemed to elicit somewhat worse outcomes in the Kaplan-Meier survival curves. Therefore, we cannot entirely dismiss that statistically significant differences could exist if larger series with more relapse cases were analyzed. Interestingly, no differences regarding gender were observed in the previously reported series, although women had appreciably worse outcomes than men in the Cox regression analysis, differences that were close to the statistical significance limit (*P* = 0.056).

Classical laboratory parameters, such as ESR and C-RP, were not valid to discriminate between those patients with or without infection relapse when determined at baseline in our series. However, the value of their sequential determination during follow-up has been described in children with acute hematogenous osteomyelitis and also in adults [13, 14, 33, 34].

Our study has several limitations, including those related to the retrospective studies, the relatively reduced sample size to analyze subgroups and the long period of inclusion, almost unavoidable in a single-center series. However, the sample size was the highest among the main series reviewed regarding the number of patients with long bone osteomyelitis from one single center, and large enough to detect significant differences in several relevant parameters. On the other hand, the single-center series ensured a greater homogeneity in the evaluation and management of the patients, the very long period of follow-up ensured the detection of late relapses and, finally, the long period of recruitment did not seem to bias the results as the year of diagnosis did not have any significant influence on the outcome (*P* = 0.8).

From our series, limited exclusively to long bone osteomyelitis in adults, we conclude that symptoms present for longer than 3 months, bone exposure and treatment other than surgical debridement with free or pedicled flaps are risk factors for osteomyelitis relapse. Therefore, the other modalities of medical or surgical treatment analyzed should be considered suboptimal. Bone exposure and duration of osteomyelitis favor poorer outcomes, regardless of the type of treatment used, constitutes therefore easy-to-detect markers of severity, and should be considered in any patient with long bone osteomyelitis. Further studies are needed to validate these severity factors and to design treatment strategies focused on a better management of these especially difficult-to-treat cases.

## Conclusions

Osteomyelitis extending for > 3 months, bone exposure and treatment other than surgical debridement with muscular flap are risk factors for osteomyelitis relapse.

## Abbreviations

CI: Confidence interval
C-RP: C-reactive protein
CT: Computed tomography (CT)
ESR: Erythrocyte sedimentation rate
MRI: Magnetic resonance imaging
MRSA: Methicillin-resistant (MRSA)
MSSA: Methicillin-susceptible *Staphylococcus aureus*
ROC: Receiver operating characteristic curve
